# Data Fusion of Observability Signals for Assisting Orchestration of Distributed Applications

**DOI:** 10.3390/s22052061

**Published:** 2022-03-07

**Authors:** Ioannis Tzanettis, Christina-Maria Androna, Anastasios Zafeiropoulos, Eleni Fotopoulou, Symeon Papavassiliou

**Affiliations:** School of Electrical and Computer Engineering, National Technical University of Athens, 10682 Athens, Greece; gtzanettis@mail.ntua.gr (I.T.); andronaxm@netmode.ntua.gr (C.-M.A.); efotopoulou@netmode.ntua.gr (E.F.); papavass@mail.ntua.gr (S.P.)

**Keywords:** observability, distributed tracing, microservices, distributed applications, edge computing orchestration, exemplars

## Abstract

Nowadays, various frameworks are emerging for supporting distributed tracing techniques over microservices-based distributed applications. The objective is to improve observability and management of operational problems of distributed applications, considering bottlenecks in terms of high latencies in the interaction among the deployed microservices. However, such frameworks provide information that is disjoint from the management information that is usually collected by cloud computing orchestration platforms. There is a need to improve observability by combining such information to easily produce insights related to performance issues and to realize root cause analyses to tackle them. In this paper, we provide a modern observability approach and pilot implementation for tackling data fusion aspects in edge and cloud computing orchestration platforms. We consider the integration of signals made available by various open-source monitoring and observability frameworks, including metrics, logs and distributed tracing mechanisms. The approach is validated in an experimental orchestration environment based on the deployment and stress testing of a proof-of-concept microservices-based application. Helpful results are produced regarding the identification of the main causes of latencies in the various application parts and the better understanding of the behavior of the application under different stressing conditions.

## 1. Introduction

With the introduction of various cloud-native technologies and the advent of micro-services-based development paradigms for cloud and edge computing applications, modern applications are becoming more and more distributed [1,2]. The term distributed refers here to applications that are broken down into a set of components (or microservices) that work together, while each component is managing a specific part of the application’s business logic. By taking advantage of virtualization and containerization techniques, each application component is packaged in the form of a container and can be independently deployed and managed. Distributed applications are usually deployed over distributed programmable compute and network infrastructure across the compute continuum, from Internet of Things (IoT) to edge and cloud computing resources. In some cases, an application may cross different types of infrastructure, platforms and technologies. The environments that host such applications tend to be dynamic, introducing a set of challenges for performance monitoring, quality of service (QoS) assurance and conformance to service level agreements (SLAs) [2].

One of the main challenges in optimally managing distributed applications is related to the need to move from traditional monitoring tools to modern observability tools [3,4]. Traditional monitoring tools do not consider distributed microservice environments and container-based interactions since they mainly target monolithic applications. Furthermore, although tracing techniques are used traditionally by software developers to track metrics related to the behavior of an application, such techniques are not designed for microservices-based applications and do not consider the capabilities of applications for horizontal scaling. To assess the performance of application components of distributed applications, there is a need for observability tools that are able to monitor the interactions among such components. The concept of cloud-native observability is emerging to provide indicators of the health and status of applications inside cloud-native elements such as containers, microservices and orchestration tools [5,6]. Such a need is also evident in the case of serverless architectures and applications, where dynamic application workloads lead to horizontal scaling of the compute resources across the compute continuum, and pose advanced requirements with regard to observability [7].

However, modern observability approaches must consider the need for integration with existing or emerging monitoring tools [4,6]. Nowadays, there is a lack of built-in observability in existing orchestration platforms for cloud-native applications. Several open-source container orchestration platforms have been made available (e.g., Kubernetes, Docker Compose), where monitoring mechanisms focus on the management of the deployment of the distributed applications and the usage of resources per application component. Such mechanisms are not intended to support sophisticated monitoring, targeted to the nature of distributed applications and the observation of metrics related to the interaction among application components. Furthermore, various third-party tools exist for supporting distributed tracing and logging mechanisms, though without a good level of integration with the aforementioned monitoring tools.

In the work presented in this paper, we aim to tackle the challenge of the fusion of data coming from orchestration platforms, distributed tracing and logging tools. The main objective is to reduce the complexity for system administrators and software developers in going through a series of monitoring data and logs to assess the performance of distributed applications. From this perspective, the main contribution of our work in this paper regards the specification of an open data interlinking scheme to support the collection and fusion of data related to the usage of resources per container (e.g., CPU/memory usage per container), service performance metrics (e.g., number of served requests per second), distributed tracing metrics (e.g., latency on serving a request, latency in the interaction between two microservices) and logs (e.g., error caused due to a container crash). To achieve this, the proposed data scheme is able to fuse and correlate data coming from different types of signals, namely metrics, exemplar metrics (similar to metrics with the addition of metadata), logs and traces. In this way, we can enable the realization of the analysis of complex microservices-based applications and the provision of useful insights into the behavior of the system, including the sources of latency. Continuous profiling of distributed applications in production can be supported, with only a small overhead from the applied distributed tracing and logging mechanisms.

Furthermore, we demonstrate how the proposed open data scheme can enable software developers and system administrators to implement solutions to collect and analyze data coming from different orchestration, logging and tracing tools. We provide details for the implementation of such a solution based on a set of open-source tools. These tools are based on the Prometheus monitoring engine [8], which is supported by the Kubernetes orchestration platform, the Zipkin distributed tracing tool [9], the Fluentd logging software [10] and the adoption of the Prometheus Python instrumentation library for the definition of exemplars in the source code [11]. Analysis of the collected data upon the deployment of a developed IoT distributed application over programmable infrastructure was performed, validating the suitability and the effectiveness of the proposed modern observability approach.

The structure of the remainder of the paper is as follows. In Section 2, we provide a short overview of the basic terms used for the description of various signals in an orchestration environment, while focusing on the distributed tracing part. In Section 3, we present related work in the field considering modern observability approaches that include distributed tracing mechanisms and their integration with emerging cloud and edge computing orchestration platforms. In Section 4, we detail the proposed orchestration-based observability approach. We present a generic data fusion schema that can be adopted and instantiated in multiple orchestration environments, along with its development in an indicative orchestration environment built upon open-source tools. In Section 5, we provide details of a distributed IoT application that was developed for experimentation purposes, the experimentation testbed and the overall validation and performance evaluation results achieved. Section 6 concludes the paper with a discussion of the main work presented, the results produced and a set of open future research areas.

## 2. Background

### 2.1. Theory and Definitions

Prior to delving into details regarding the available work in the area of modern observability techniques and our proposed approach, we consider it helpful to provide short definitions of the main terms used.

#### 2.1.1. Signals, Observability and Instrumentation

**Figure 1:**
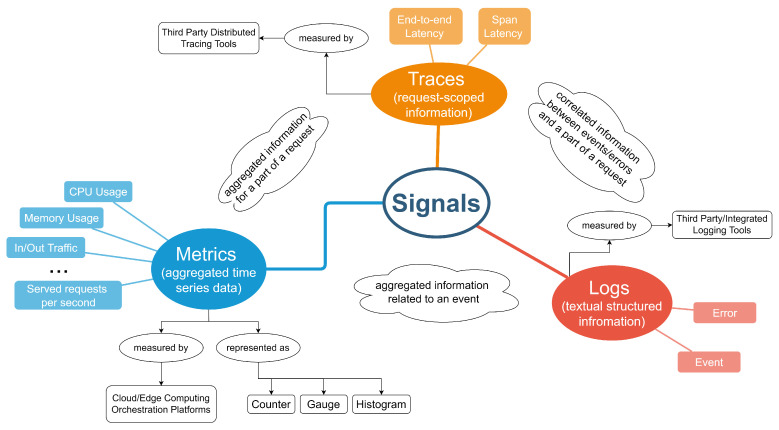
Classification of signals into metrics, logs and traces.

Initially, we clarify the concept of observability by borrowing the definition provided in the area of control theory. According to control theory, observability is a measure of how well the internal states of a system can be inferred from knowledge of its external output [12]. It involves the collection, visualization and analysis of a set of signals. In the case of distributed monitoring and orchestration mechanisms, these signals can be classified into metrics, logs and traces. Their holistic consideration and correlation helps system administrators and software developers to identify and better understand the causes of errors or malfunctions in the provided software (see Figure 1). In this way, observability moves one step forward compared to monitoring mechanisms, since it can provide information regarding the causes of the errors and enable their resolution.

Metrics refer to data that are based on numeric representations of state at a specific timestamp or during a period of time (aggregated numeric representation). They are usually used for first-response alerting and decision making in an orchestration system (e.g., rule-based systems for autoscaling based on threshold values [13]). Metrics can be represented through a counter (e.g., incoming HTTP requests), a gauge (e.g., the current depth of a queue) or a histogram (e.g., duration of a request). Typical examples of metrics include resource usage metrics (e.g., CPU or memory usage), traffic served (e.g., incoming or outgoing traffic per second) and requests served (e.g., HTTP requests served per second). The vast majority of metrics can be made available through monitoring components of cloud and edge computing orchestration platforms.

Logs refer to structured information regarding discrete events. Such information is made available in a textual structured representation and can be easily interpreted by humans. Logs usually describe usage patterns, events, activities and operations within an orchestration system (e.g., application debug or error messages). Information collected through a set of logs can be combined to provide insights regarding situations or events. Logs are usually made available through third-party tools that are interoperable with cloud and edge computing orchestration platforms.

A distributed trace regards a set of operations that represent a unique transaction handled by the application. Thus, traces can be mapped with a request scope. The transaction/request actually includes a flow of operations that are provided across the microservices of the application. By examining distributed traces, we can more easily understand what happened during a distributed transaction and identify the existence of delays or bottlenecks in the overall flow. Typical examples of information provided through distributed tracing regard latencies in the execution of software within a microservice, the interaction between microservices and end-to-end latencies for serving a specific request. Distributed tracing information is made available through third-party tools with a low level of integration and interoperability with cloud and edge computing orchestration platforms.

Observability considers the combination of these types of signals (metrics, logs, traces). In the selection of the set of signals to be monitored, the trade-off between the availability of rich information and performance or complexity aspects must be considered. Upon identification of the set of signals, the collection of the relevant information is enabled based on proper instrumentation of the deployed software. In the case of distributed tracing especially, instrumentation plays an essential role since it implements the monitoring interfaces for the collection of information related to the latencies that appear per trace. In this case, the instrumentation is request-scoped, in accordance with the needs of proper monitoring of cloud-native distributed applications.

Moving one step further, correlations among the information collected by the various signals must be supported; however, this is not an easy task. It is especially complex in large distributed applications consisting of multiple microservices that handle numerous requests. For instance, as depicted in Figure 1, correlation between metrics and traces can be realized on a request basis where aggregated time series data can be attributed to a specific part of the trace. Similar correlation can take place between metrics and logs, while logs and traces can be also correlated based on aggregated events that are associated with a specific part of a trace.

To enable this kind of correlation among the signals, the concept of the exemplar is introduced as an extra signal type. An exemplar is considered to be a metric that can hold both metric values and further metadata in the form of labels. The labels can include the request_id or trace_id defined by the applied distributed tracing mechanisms. Each value of a metric exemplar is also associated with a timestamp. In this way, information provided through exemplars can act as a glue for correlating information coming from pure metrics, logs and traces, mainly based on the joint trace_id and timestamp values. Exemplars can be used for monitoring the numbers of certain errors, as well as latency values and request ratios in the various microservices and traces. Exemplars facilitate the interlinking of aggregated metrics in the relevant situation.

**Figure 2:**
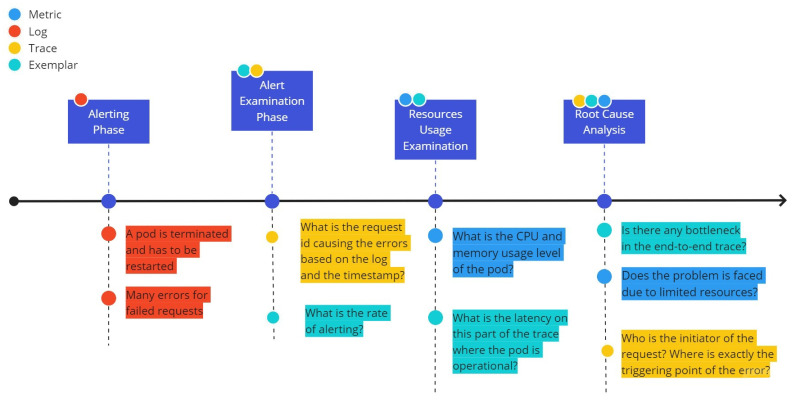
Exemplar usage example.

An example is provided in Figure 2. In this example, alerting information related to bad behavior of a container that is hosting a microservice of a distributed application, combined with numerous errors for failed requests from this container (alerting phase), is associated with the id of the trace that is related to the failure and the rate of failures, which is supported by an exemplar for this trace ID (alert examination phase). Subsequently, examination of the usage of resources in this container takes place to examine the need for assigning further resources to tackle the problem, while the latencies in the various parts of the trace are also examined (resources usage examination). As we are able to correlate the aforementioned signals, we can proceed to a root cause analysis, shed some light on identifying the main cause of the problem and undertake corrective actions.

#### 2.1.2. Distributed Tracing Terminology

In this section, we delve into a detailed description of the main concepts used in distributed tracing mechanisms. The term distributed tracing refers here to a method of profiling and monitoring applications built using a microservices-based architecture. It is a type of correlated logging that helps to gain visibility into the operation of a distributed software system [14], pinpoint where failures occur and identify the causes in cases of poor performance. Through the collection of distributed traces, a complete analysis of the performance of a distributed application can take place, including root cause analysis of failures, performance profiling and debugging (identifying inefficient code) in production. Distributed tracing helps software developers to quickly understand the flow of requests through the microservices that make up a distributed application.

**Figure 3:**
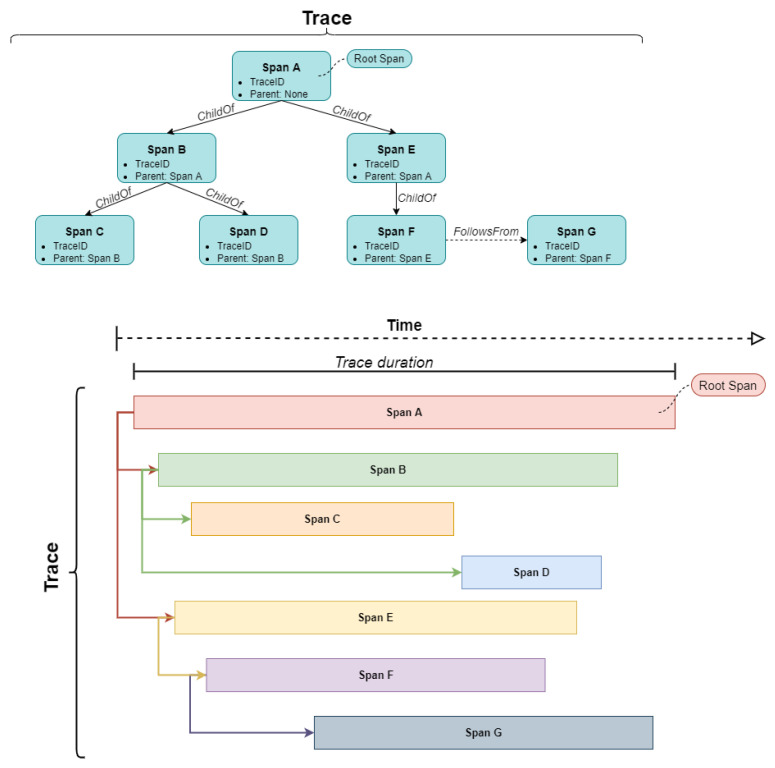
Distributed trace structure.

A distributed application may have a set of distributed traces. As already mentioned, each trace is related to a set of operations that represents a unique transaction. Each one of the operations is defined as a span of the distributed trace. According to the OpenTelemetry specification [15], a trace can be thought of as a directed acyclic graph (DAG) of spans, where the edges between spans are called references. Each span has a descriptive name related to the operation that it supports, a start and end time, a set of tags (optional field) for collecting additional information and a span context that encapsulates implementation details. The definition of the spans that constitute a trace follows a hierarchical approach, allowing the assembly of multiple spans into a complete trace [15]. Different types of relationships may be defined between spans. A span may reference zero or more other span contexts, based on two types of references, namely the ChildOf and the FollowsFrom references [15]. In the ChildOf reference type, a parent (or root) span may include a set of child spans. In the FollowsFrom reference type, a sequential execution of spans can be defined. By considering both reference types, a specific path is created for showing how a particular transaction is executed through the numerous components that make up the application (Figure 3).

## 3. Related Work

Various research studies and surveys are emerging in the area of observability mechanisms that include distributed tracing techniques, focusing mainly on the exploitation of the collected information for analysis purposes. Based on an industrial survey on microservice tracing and analysis [16], distributed tracing and analysis is considered an important part of the infrastructure for industrial microservice systems, while the development of efficient data fusion mechanisms for trace analysis and production of business intelligence reports is considered challenging. In a similar qualitative study on identifying the challenges and good practices in the field of observability and monitoring of distributed systems [17], the main reported challenge was found to be the need for the management of heterogeneity in the adopted microservices-based development and deployment paradigms, since isolated monitoring and observability solutions are adopted even across teams within the same organization. Targeted studies on specific development paradigms are also provided, such as the study in [7] that focuses on testing and debugging of serverless-based applications. In this case, distributed tracing techniques are proposed to analyze how a call propagates through different services, functions and resources and to see transactions as a flow of many lambda functions.

Several approaches are also available for supporting automated data analysis over instrumentation frameworks. In [18], Pythia is presented as an automated framework that suggests the activation of instrumentation tools to manage a newly-observed performance problem. In [19], the authors propose a solution for correlating information that is present in multiple traces, considering all traces as a single graph and decomposing tracing data into multiple vertices and edges. In [20], OpenTelemetry traces are processed to identify bottlenecks in the performance of a distributed application that is deployed across the cloud continuum. A data-centric perspective on tracing is detailed in [21], where the authors implement three different approaches to data-centric distributed tracing in a distributed data processing system built using microservices, and discuss their advantages and disadvantages. In the work presented in [22,23], tracing data are used to extract service metrics, dependency graphs and workflows with the objective of detecting anomalous services and operation patterns. As stated in [23], timely and accurate detection of trace anomalies is very challenging due to the large number of underlying microservices and the complex call relationships between them. In addition to anomaly detection, distributed tracing techniques are applied for privacy risk detection in [24], where a framework is introduced to identify privacy and security risks associated with the dissemination of data through the path a service request follows.

Research work is also emerging in the area of development of novel observability frameworks for assisting orchestration actions. In [25], an observability framework for microservices orchestration is detailed, utilizing a cloud-based infrastructure, that provides the means to understand the internal behavior of microservices at different layers and different lifetime and abstraction levels. These layers are associated with data collection and retrieval, raw data storage, data processing and correlation and data visualization and alerting. Multilevel observability is introduced in [26], considering both application-oriented and infrastructure-oriented metrics to improve automatic orchestration in cloud environments. The use of distributed tracing techniques to support observability in serverless applications and provide insights for troubleshooting purposes is examined in [27]. In [28], an approach is provided to evaluate observability of microservices both in an offline and an implementation-agnostic manner. Intra- and inter-service execution paths are described as a behavioral model of the microservices under observation, serving as input to an application workload generator that produces realistic trace data. In [29], observability is considered as one of the main desirable features for supporting autonomic management of cloud-native applications. In [30,31], a service-mesh approach for supporting observability features is adopted to avoid custom instrumentation of each application based on the use of specific software libraries.

In parallel, a set of open-source distributed tracing solutions are emerging under the umbrella of cloud-native solutions, as managed by the Cloud Native Computing Foundation (CNCF) [32]. To name only a few, these include Zipkin [9], Jaeger [33], Elastic APM [34] and Apache SkyWalking [35]. Most of the emerging tools follow the OpenTelemetry specification [15], which is a collection of tools, APIs and SDKs used to instrument, generate, collect and export telemetry data (metrics, logs and traces) to help software developers to analyze the software performance and behavior.

An overview of a set of well-known tools for distributed tracing solutions is provided in Table 1, considering their licensing schemes, the types of signals that they observe, the main functionalities and analysis types that are supported, their compatibility with evolving specifications and their popularity. It is evident that a wide range of tools are under continuous development for tackling observability challenges. However, to the best of our knowledge, none of the existing open-source tools supports an open data scheme for data fusion purposes, able to make the collected data available to end users for analysis purposes.

## 4. An Orchestration-Based Observability Approach

The approach proposed in this paper aims to support data fusion of observability signals in distributed applications. These applications are deployed by cloud and edge computing orchestration platforms over programmable resources in the compute continuum. We consider different types of signals, as detailed in Section 2.1.1. In Section 4.1, we present an open and generic data schema that can be used for data fusion of the various types of signals. The data schema has a proper level of abstraction and is generic enough to support its easy integration into existing and emerging orchestration solutions for edge and cloud computing applications management. To validate its proper design and its applicability, in Section 4.2 we provide details for the development of an observability framework in a real orchestration environment, based on the use of the proposed data schema. It should be noted that the presented development is indicative and can be easily replicated based on the use of alternative observability and orchestration tools.

### 4.1. Generic Data Fusion Schema

**Figure 4:**
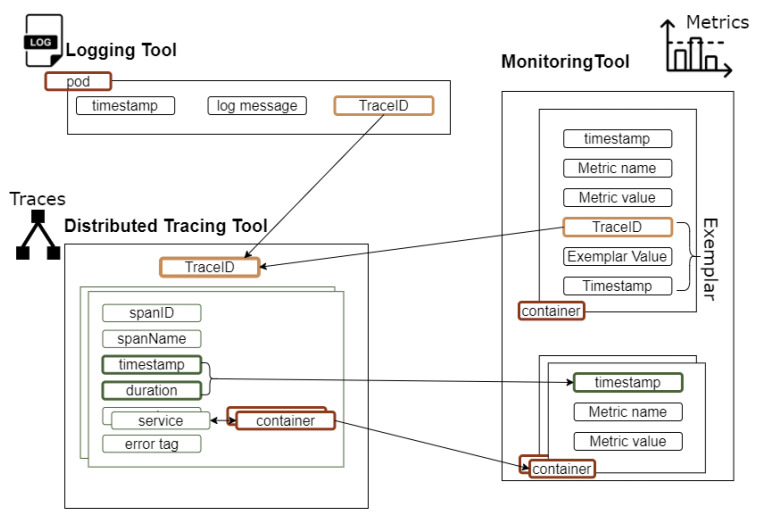
Data fusion schema.

A data schema has been designed aiming to enable the correlation of information coming from the different types of signals, as depicted in Figure 4. We consider the management of a distributed application consisting of multiple traces, where each trace is broken down into a set of spans. A span is associated with a microservice (or a part of a microservice) and is executed within the container that is hosting the microservice.

Starting with the distributed tracing tool, we are interested in tracing data where, for each trace ID, we collect information for the set of span IDs that are associated with it. For each span ID, we collect information for the microservice that it supports, the timestamp and duration of the execution of the span and the container where the microservice is hosted. Given that every piece of the collected data is associated with a timestamp, timing information enables us to filter data for signals within a certain time window.

Based on the information for the container and the timestamp, we can enrich the dataset with information provided in the form of metrics. These include deployment metrics given by the monitoring mechanisms of the orchestration platform, exemplars and application metrics provided through the instrumentation library. In the case of the deployment and application metrics, information related to the metric name and value in each container is associated with the relevant information from the distributed tracing tool. In the case of exemplars, correlation is also supported through the metadata that accompany each metric, including the trace ID.

Similarly, based on the trace ID and the container, logging information can be associated with the information provided by both the distributed tracing tool and the monitoring mechanisms. The critical point here is to ensure that the ids reported by the logging and the tracing systems are exactly the same. In this way, navigation across information collected through logs and traces and the associated individual request can take place.

### 4.2. Observability Approach within an Orchestration Environment

**Figure 5:**
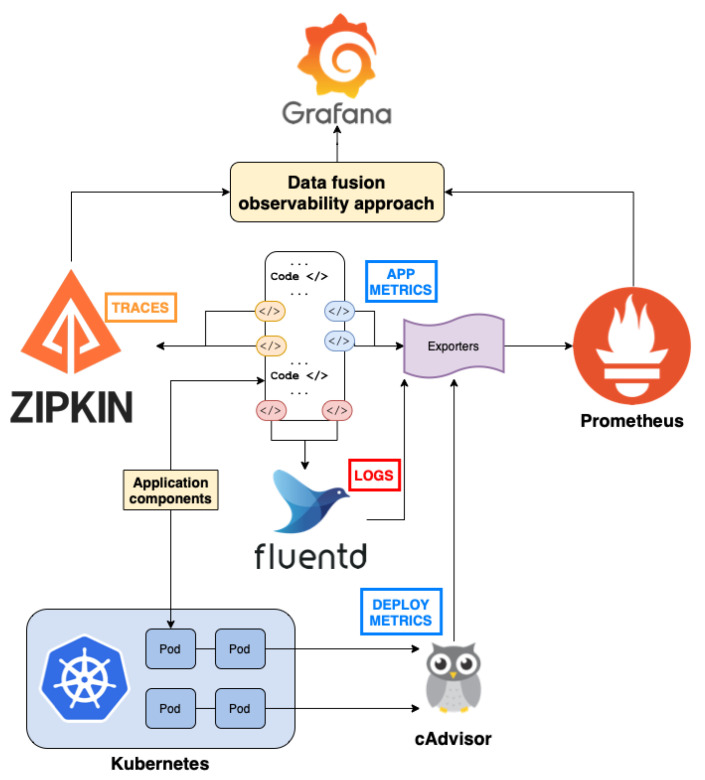
Instrumentation architecture.

Based on the data fusion schema presented in Section 4.1, we proceeded to the design and deployment of an orchestration environment able to provide data that can be represented through it. The orchestration environment is based on the Kubernetes orchestration platform for distributed applications, as depicted in Figure 5. The data collected can be further categorized into two parts, one concerning signals received from the application itself such as traces, logs and application metrics, and another concerning metrics collected by the utilized resources, such as CPU and memory usage.

Five main open-source tools were used and integrated with the Kubernetes-based orchestration environment for supporting the collection of observability signals. The deployment metrics were provided by the monitoring components of the orchestration platform and specifically the Prometheus monitoring engine [8]. Prometheus provides time-series data for a variety of metrics, including CPU usage, memory usage and incoming and outgoing traffic per pod. Resource usage and performance metrics are made available to Prometheus through cAdvisor. cAdvisor is a framework that has been efficiently integrated with Kubernetes, providing multiple metrics concerning the pods and containers that are used during the lifetime of the application, and this was exploited for adding deployment metrics to our overall data schema.

Exemplar metrics were made available based on the use of an instrumentation library during the development of the distributed application. The exemplar metrics are associated with the measurement of specific request latencies and error rates, based on the proper instrumentation probes introduced in the source code. Instrumentation libraries are available in various programming languages including Go, Python and Java. In our case, we used the Prometheus Python client [11], where exemplars can be added to counter and histogram metrics.

Traces awere collected based on the Zipkin distributed tracing tool [9]. For tracing latency metrics and interactions between application components, we created a trace for each request received outside the application, including a tree structure of spans within the functionality of each trace. To keep track of this trace/span structure, unique trace/spans IDs were used, while parent span IDs were also used to preserve the hierarchy between the spans.

Logging was supported based on the Fluentd logging software [10]. Fluentd supports a unified logging layer where different types of logs (e.g., application errors, warnings) are represented through a JSON format. Fluentd allows various logs to be collected from a Kubernetes cluster, processes them, and makes them available in monitoring tools such as Prometheus. Grafana was also used as a main dashboard for visualizing information coming from the different types of signals [36]. Grafana pulls observability measurements from all the aforementioned tools according to the data fusion schema, to draw together meaningful plots of the correlated signals. These can be exploited by the developer to understand the relations between the heterogeneous observations and to facilitate the initiation of additional and/or deeper analysis.

It should be noted that, although specific tools were selected for supporting widely popular open-source monitoring, tracing and logging frameworks, the proposed data fusion scheme is generic enough to be applicable in different types of orchestration environments. Different languages and the associated instrumentation libraries can be selected, while the backend storage solutions for accessing the collected observations can also be differentiated. Depending on the selected tools, parameters related to the collection of the measurements can also be fine-tuned (e.g., Zipkin instrumentation allows a variable sampling rate). High levels of configurability are also supported in terms of the set of monitored signals. For instance, deployment metrics similar to the CPU and memory usage of the containers that support specific parts of the application can be monitored. Similarly, different application and log metrics can be considered, such as byte rates, request queue overflows and bad request error codes. The exact specification of the signals to be observed is the responsibility of the software developer and/or the system administrator in each deployment.

Based on the data fusion schema presented in Section 4.1 and the detailed implemented observability approach developed around the Kubernetes orchestration environment, we have produced a JSON schema for representing the collected and fused data. The structure of the JSON schema is shown in Listing Section 4.2. The proposed JSON schema includes joint information coming from the Zipkin distributed tracing tool (information per trace ID and span_id related to the duration of the spans and the pods where they are executed), information coming to Prometheus through exemplars (information per trace ID related to the observed latencies per span or trace), information coming to Prometheus from the cAdvisor tool (CPU and memory usage per pod) and information coming from the Fluentd logging system (error messages per trace or span ID).

**Listing 1.** JSON data fusion schema in Kubernetes orchestration environment.

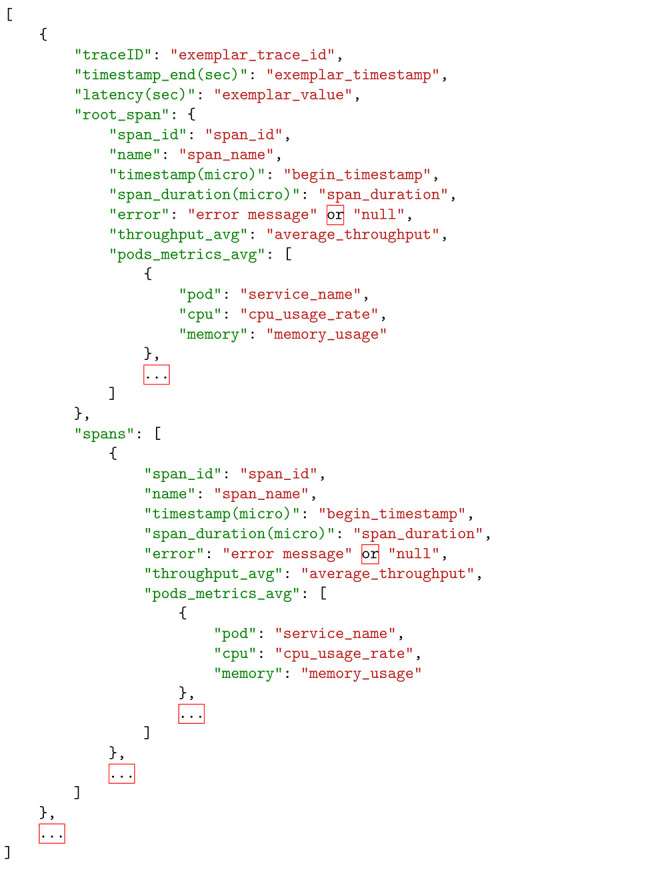



### 4.3. Assisting Orchestration Mechanisms

Upon the fusion of data coming from the various observability signals, assistance in decision making for taking orchestration actions can be provided [26]. Such assistance can be given to system administrators based on the interpretation of results or provided in an automated manner based on the design of intelligent orchestration mechanisms, usually via the exploitation of artificial intelligence (AI) techniques. Deployment and scaling policies can be developed taking into account the resource usage profiles of the application components that compose an application graph. This is especially helpful in cases where high resource usage is associated with increasing latencies in the serving of requests in a specific span or in the overall trace. Co-location of a set of application components of an application graph may be also requested in cases of identifying increased latencies in the interaction among specific components that can be due to congestion in the networking part of the infrastructure. Security alerts can be also triggered in cases of an increase in the error rates per application component (e.g., identification of a cyber-attack with a very bursty workload).

## 5. Distributed Application Implementation and Evaluation Results

To validate and evaluate the approach proposed in Section 4, we developed a distributed IoT application and realized a set of experiments by stress testing this application under various workloads. The developed IoT application, including the source code for the instrumentation part and the source code for supporting the data fusion mechanism, is openly available at [37]. The experiments took place over a testbed in a laboratory environment, where the tools mentioned in Section 4.2 were deployed. Specifically, a Kubernetes cluster with three nodes was set up, where each node uses a four-core Intel-based CPU, 16 GB of memory and 100 GB of disk storage space. Each application component was deployed as an independent service in the form of a Docker container within a Kubernetes pod. The communication among the components was HTTP-based and made use of the cluster’s internal network. Regarding the applied stress tests for the evaluation part, we developed two workload profiles based on the use of the Vegeta HTTP load testing tool [38].

It should be noted that the developed novel observability framework is applicable to monitoring multiple applications in parallel, as they are deployed and managed by the applied orchestration framework. However, we were interested in proceeding to a performance analysis with a per-application scope. Specifically, we aimed to examine the performance of the developed IoT application, focusing on the latencies monitored in the various traces and spans of the application and the associated usage of resources. Thus, the presented results are related to a specific deployment of the IoT application over the provided testbed, and its stress testing based on the produced workload profiles.

### 5.1. Distributed IoT Application

**Figure 6:**
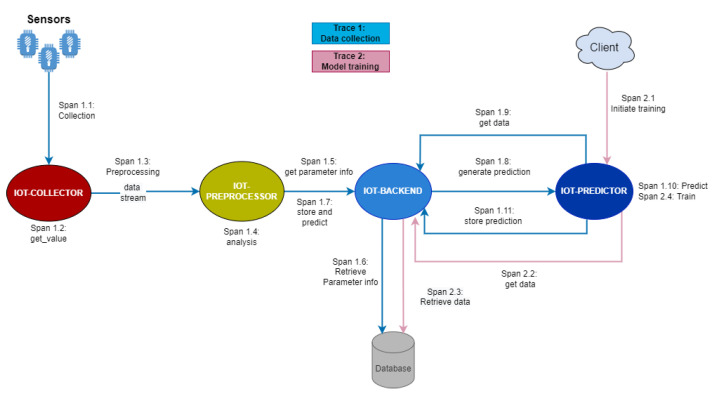
Distributed IoT application graph.

The distributed IoT application aims to collect and analyze real-time and historic data streams that are provided by IoT nodes. The application graph is depicted in Figure 6. It consists of a set of components (or microservices) that support the overall application business logic. These components are:

- Data collection component (IoT Collector): this component is located at the far edge part of the infrastructure and is directly connected to the IoT nodes to manage collection and transmission of sensor values. It can be massively replicated according to the needs of the platform (e.g., how many sensors are in place).
- Data aggregation component (IoT Preprocessor): the data aggregation component is responsible for aggregating the data collected by the IoT Collector and applying basic data management functionalities (e.g., data aggregation, data filtering, outliers removal). It can be replicated according to locality requirements and the number of IoT data streams served at each point of time. It is deployed at the edge part of the infrastructure for guaranteeing latency requirements based on processing of data close to the location of the IoT nodes.
- Backend database (IoT Backend): this is associated with the database system that stores the data streams for offline analysis, model training, etc. This component is central to the overall architecture and is deployed in the cloud part of the infrastructure. It can be scaled up and down according to the server workload profile.
- Data analysis—Forecasting (IoT Predictor): this component is responsible for data analysis of the collected time-series data. It can be scaled according to traffic and data volumes. For the purpose of this analysis, a long short-term memory (LSTM) model is used for predicting future values of the time-series data.

### 5.2. Traces Analysis

**Figure 7:**
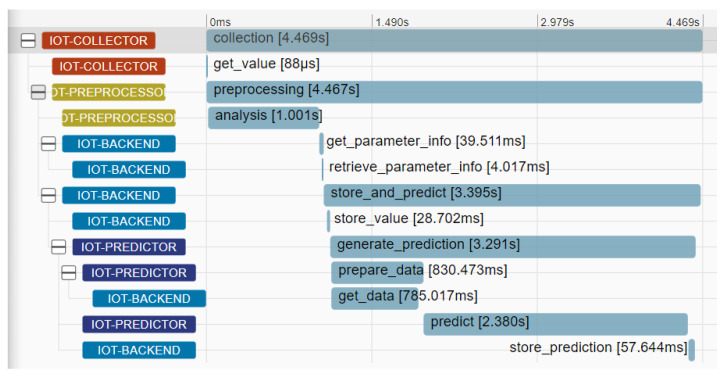
Trace 1 analysis in Zipkin.

Two main traces of the distributed IoT application have been identified and selected for analysis purposes, as depicted in Figure 6. The first trace (trace 1, consisting of the spans 1.1 to 1.11) supports the flow of real-time data collection and analysis. Data are collected from the IoT nodes (spans 1.1 and 1.2), pre-processed (spans 1.3 and 1.4) and stored in the database (spans 1.5 and 1.6). Subsequently, they are sent for analysis (spans 1.7 to 1.10), while the analysis results are also stored in the database (span 1.11). An indicative analysis of this trace through the Zipkin distributed tracing tool is depicted in Figure 7. It can be seen that the largest spans in terms of introduced latencies in the application part are the spans 1.4 (pre-processing data analysis) and 1.10 (prediction process).

**Figure 8:**
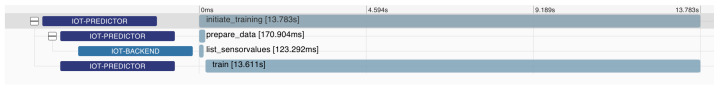
Trace 2 analysis in Zipkin.

The second trace (trace 2, consisting of the spans 2.1 to 2.4) supports the flow of historic data analysis, where an end user is requesting the offline retraining of the model using the historical data collected up to this point. The end user initiates a training request (span 2.1), the data for the training processes are fetched (spans 2.2 and 2.3) and the training process is executed (span 2.4). An indicative analysis of this trace through the Zipkin distributed tracing tool is depicted in Figure 8. As expected, the largest span in trace 2 is span 2.4, where the training process takes place.

### 5.3. Validation and Performance Evaluation

**Figure 9:**
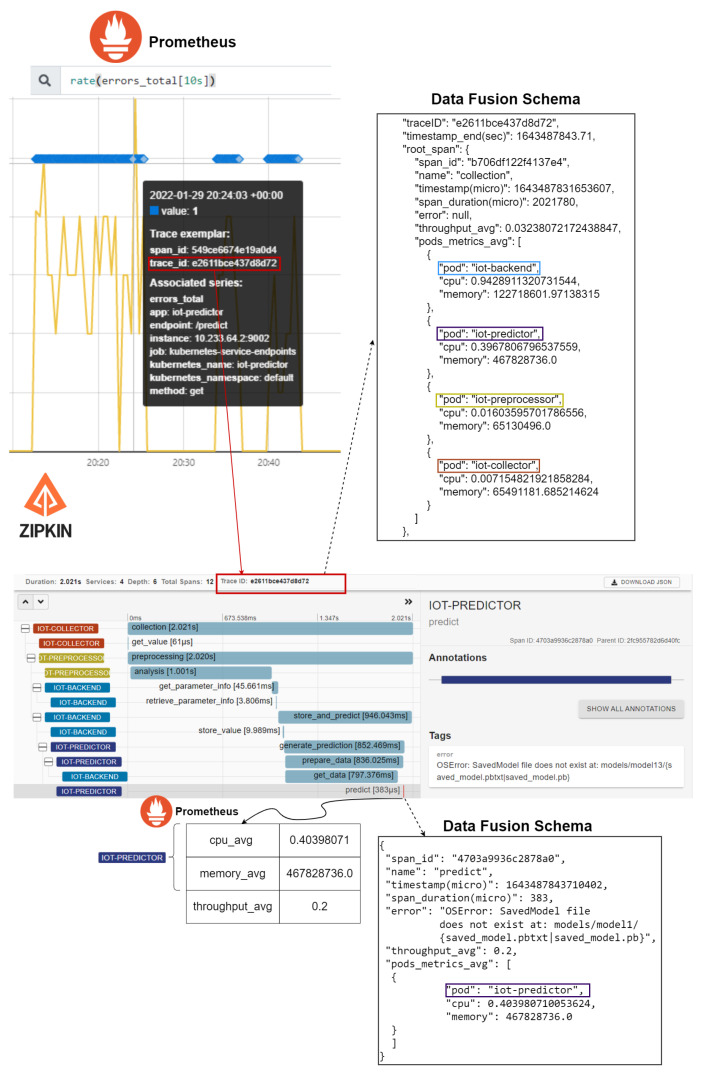
Integrated visualization capabilities in the orchestration environment.

In this section we present a set of validation and performance evaluation results that were produced based on the realization of a set of experiments over the implemented observability approach and based on the use of the developed distributed IoT application. Prior to delving into the details of the realized stress tests and the produced results, in Figure 9 we provide a high-level view of the integrated visualization capabilities based on the supported fusion of the collected data.

Starting with the production of an error log for the IoT predictor component, we wish to examine in detail what occurs on the specific trace and to identify the source of the error (e.g., whether it is due to a performance issue or a misconfiguration). Through the use of exemplars, the error rate for a specific component can be visualized, providing an overview of the frequency and the timing of the error production. Based on the ID of the trace under analysis, we can examine the introduced latencies per span through the Zipkin visualization tool and identify any irregular behavior. Moving one step further, through the trace ID and the pods identification, we can examine the performance of the IoT predictor component in terms of the error production rate (monitored in Prometheus through the use of exemplars) and the associated usage of resources from this component in the pod where it is deployed (relevant metrics in Prometheus). A holistic analysis can take place and lead to meaningful insights for the system administrators and/or the application developers, similar to the conceptualization that was presented in Figure 2.

Next, we present evaluation results based on the processing of two different types of workloads by this trace (trace 1 in the analysis in Section 5.2) for the distributed IoT application. The first workload follows a smooth increase and decrease in the number of requests to be served, based on the availability of new IoT node readings. The second workload follows a more bursty behavior, based on the sudden availability of multiple sensor readings at the same time, with the appearance of two spikes during its execution. Considering a basic rate λ for the submission of requests, the first workload follows the rates (λ–3λ–5λ–3λ–λ), while the second workload follows the rates (λ–5λ–λ–5λ–λ). Both workloads were prepared based on the use of the Vegeta stress testing tool [38].

**Figure 10:**
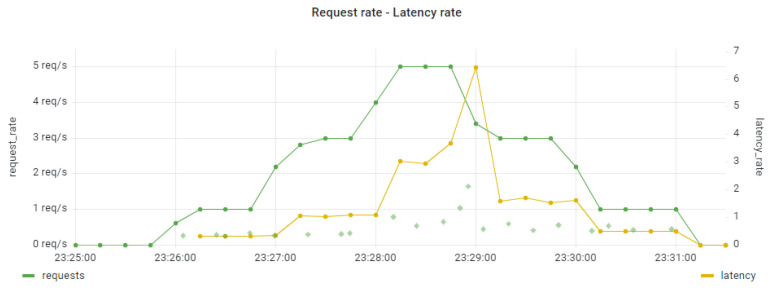
Request rate and latencies variation under the smooth workload.

**Figure 11:**
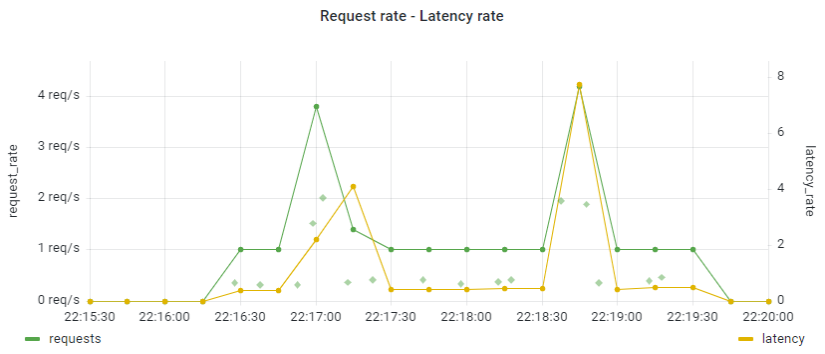
Request rate and latencies variation under the bursty workload.

In Figure 10 and Figure 11 we present the end-to-end latencies in trace 1 with regard to the request rate for the smooth and the bursty workload scenarios. For the latencies, we present the exact latency value based on the collection of exemplar metrics (as shown by the grey dots) and the latency rate (as shown by the yellow line). In both the smooth and the bursty workload, it seems that the end-to-end latencies follow the trend of the relevant workload, with a small delay in the appearance of spikes. This is not a desirable behavior, given that the change in the values of the latency may have a direct impact on the overall QoS of the provided application. A potential corrective action involves the application of a scaling policy for part of the application graph components (or microservices). Further examination of the main source of latency across the various spans of the trace is required. This is possible, since through the use of exemplars we obtain information for the exact trace ID for each latency value, and we can examine in detail the relevant request within the tracing tool.

**Figure 12:**
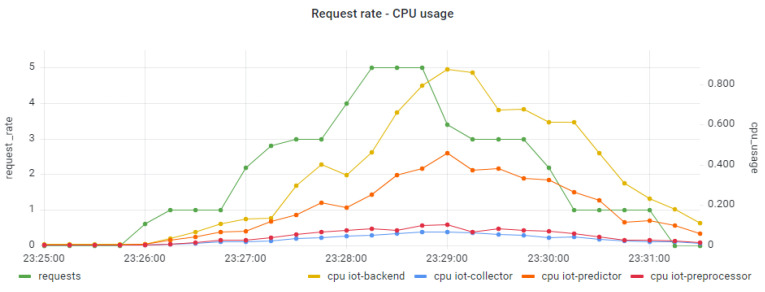
CPU usage per application component under the smooth workload.

**Figure 13:**
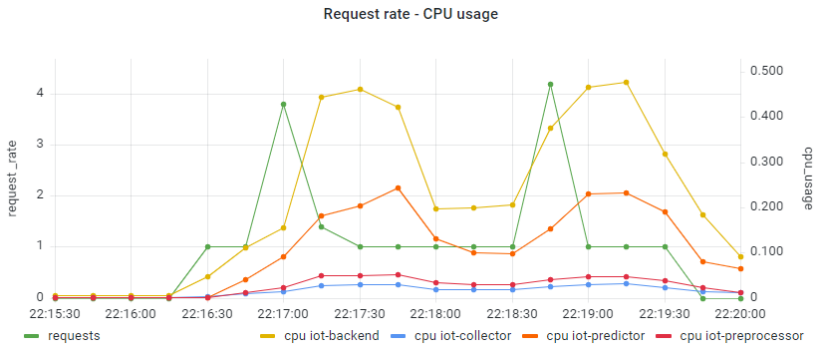
CPU usage per application component under the bursty workload.

**Figure 14:**
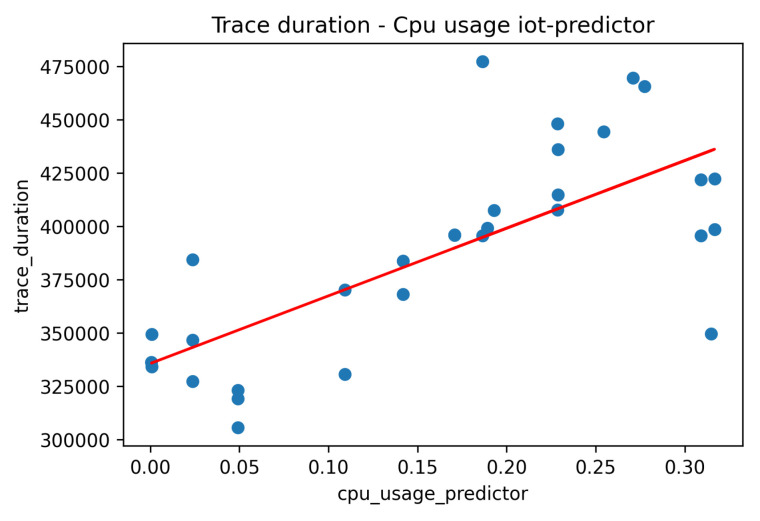
Correlation between trace duration and CPU usage—IoT Predictor component.

Moving one step further, we examine the resource usage (CPU, memory) trends per component of the distributed IoT application. It should be noted that, in our case, each pod is managing one application component and is packaged in one container. In Figure 12 and Figure 13 we present the CPU usage per application component with regard to the request rate for the smooth and the bursty scenarios, respectively. In both cases, it seems that the CPU usage follows the trend of the relevant workload for all the application components. Higher levels of CPU usage are seen for the IoT Backend and the IoT Predictor components, proving indications of the need to apply scaling policies in both components to better serve the relevant workload and avoid failures in the processing of requests. In the case of the IoT Backend component, scaling is mainly required due to the high usage of CPU resources (up to 80%). In the case of the IoT Predictor component, scaling is required due to the increased latency that is associated with this component, as identified in the trace analysis part in Figure 7. To confirm this statement, a linear regression model was produced for examining the correlation between the overall trace duration and the CPU usage of the IoT Predictor component, as depicted in Figure 14. Once again, the realization of such a joint root cause analysis was enabled based on the adoption of the proposed data fusion scheme and the integration of all the collected signals into a unified JSON representation.

**Figure 15:**
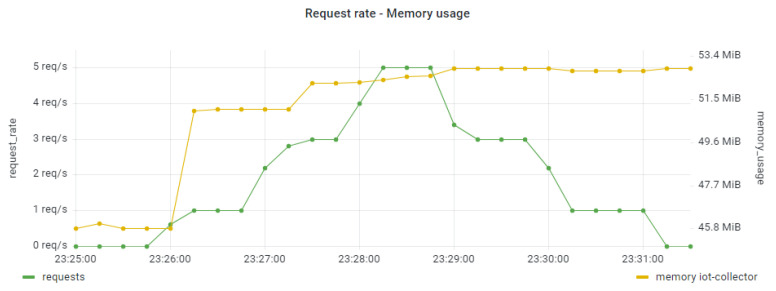
Memory usage for the IoT Collector component under the smooth workload.

**Figure 16:**
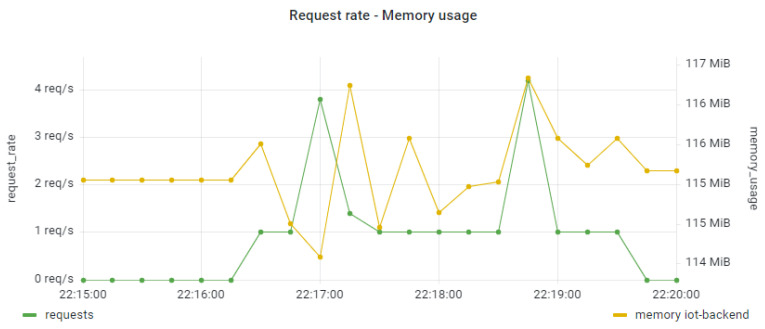
Memory usage for the IoT Backend component under the bursty workload.

**Figure 17:**
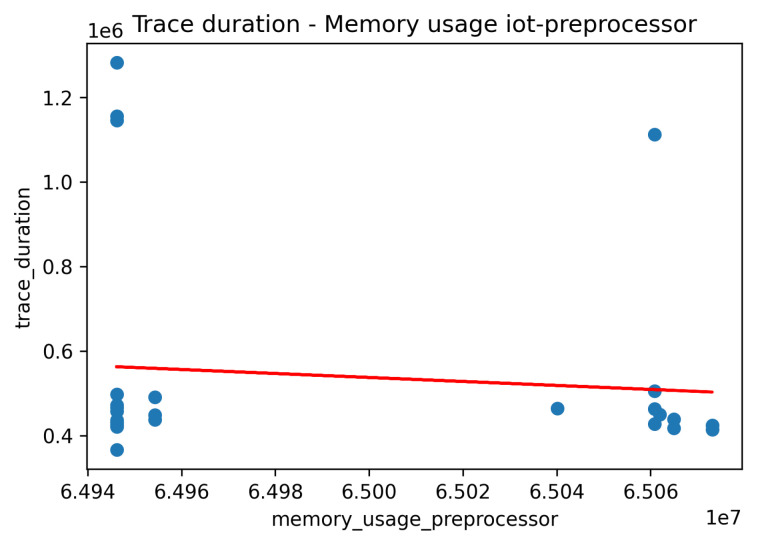
Correlation between trace duration and memory usage—IoT Preprocessor component.

Similar analysis to the CPU usage was performed for the examination of the memory usage per application component. Indicatively, in Figure 15 and Figure 16 we provide such results for the IoT Collector component (under the smooth workload) and the IoT Backend component (under the bursty workload). It seems that there was no severe impact on the memory usage in serving the relevant requests in all the examined scenarios. This can also be seen in Figure 17 in the linear regression model produced for examining the correlation between the overall trace duration and the memory usage of the IoT Preprocessor component.

Summarizing, it can be argued that through the realization of the analysis of the fused observability signals, various insights can be extracted regarding improvements that can be made at both software development and orchestration level. At the software level, the examination of high latencies in part of the components, along with the associated error logs, can provide hints to software developers to proceed to improvements in the source code. At the orchestration level, the examination of high latencies, along with the increased usage of the available resources, can provide hints to system administrators to better configure deployment and operational policies to optimally serve the relevant workload.

## 6. Discussion

In the current work, we have presented a modern observability approach based on the fusion of data signals coming from different components of a cloud or edge computing orchestration ecosystem. The approach aims to contribute to the emerging research area of cloud-native observability by providing a data fusion scheme along with a proof-of-concept implementation in a cloud-native orchestration environment based on Kubernetes. Based on the produced artifacts in the current work, an experimentation process was applied where a set of stress tests were realized to validate and evaluate the efficiency of the developed data fusion mechanisms.

It was shown that through the proposed approach, new methods of data analysis are made available to system administrators and software developers, well-suited to the domain of cloud-native computing and microservices-based applications development. Through the modern observability approach, we were able to gain a better view of how distributed applications behave under different stressing conditions and develop mechanisms to troubleshoot problems and optimally affect the application status. The overall data management effort was significantly reduced, since all the data were interlinked and made available in a common schema. Furthermore, it was shown that the approach can be easily implemented based on the use of open-source and well-supported monitoring, tracing and logging tools.

Given the recent efforts made in the development of such observability approaches, several open areas and challenges can be identified that can be tackled in the future. These include the development of tools that can easily manage, visualize and analyze the interlinked observability data, the design of AI-assisted orchestration mechanisms that take advantage of the plethora of fused data and the examination of the impact of the introduction of multi-level observability signals on the applications’ performance, based on continuous profiling of distributed applications.

## Figures and Tables

**Table 1:** Distributed tracing tools list.

Distributed Tracing Tool—Licence	Signal Types	Main Functionalities	Compatible Specifications	Popularity
Elastic APM (Open-source)	Metrics, Logs, Traces	Distributed tracing, Visual dependency mapping, Root cause analysis	OpenTelemetry, W3C trace context, Jaeger	378 Forks, 972 Stars
Pinpoint (Open-source)	Metrics, Traces	Distributed tracing with Bytecode instrumentation, Topology and data visualization	-	3.6k Forks, 12k Stars
SkyWalking (Open-source)	Metrics, Logs, Traces	Distributed tracing, Topology visualization, Root cause analysis, Service mesh	OpenTelemetry, SkyWalking agents	5.5k Forks, 18.7k Stars
Jaeger (Open-source)	Traces	Distributed tracing, Trace storage, Querying and visualization, Topology visualization	OpenTelemetry, OpenZipkin	1.8k Forks, 15.2k Stars
Zipkin (Open-source)	Traces	Distributed tracing, Trace storage, Querying and visualization, Topology visualization	OpenZipkin	2.9k Forks, 15.2k Stars
Aspecto (Commercial)	Logs, Traces	Distributed tracing, Root cause analysis, Topology visualization, Trace and logs correlation	OpenTelemetry, Jaeger	-
Honeycomb (Commercial)	Metrics, Logs, Traces	Distributed tracing, Telemetry data storage, Querying and visualization, Alerting	-	-
Lightstep (Commercial)	Metrics, Logs, Traces	Distributed tracing, Root cause analysis, Metrics and traces correlation, Telemetry data storage, Querying and alerting	OpenTelemetry, Zipkin, Jaeger	-
Grafana Tools (Tempo, Loki) (Open-source)	Metrics, Logs, Traces	Trace and logs storage and querying, Alerting functions	Zipkin, OpenTelemetry, Jaeger, OpenCensus	171 Forks, 1.9k Stars
OpenTelemetry (Open-source)	Metrics, Logs, Traces	Instrumentation tools, Programming interfaces (APIs), SDKs	Backwards compatible with OpenCensus	137 Forks, 426 Stars
OpenCensus (Open-source)	Metrics, Traces	Instrumentation libraries	-	316 Forks, 1.9k Stars

## Data Availability

Not applicable.

## Abbreviations

The following abbreviations are used in this manuscript:

AI	Artificial Intelligence
CNCF	Cloud Native Computing Foundation
DAG	Directed Acyclic Graph
IoT	Internet of Things
QoS	Quality of Service
LSTM	Long short-term memory
SLA	Service Level Agreement
